# DISPROPORTIONATE INCARCERATION AFTER THE 1994 CRIME BILL AND COGNITIVE FUNCTION IN OLDER ADULTS RACIALIZED AS WHITE

**DOI:** 10.1093/geroni/igad104.0614

**Published:** 2023-12-21

**Authors:** Paris Adkins-Jackson, Ariana Gobaud, Boeun Kim, Tiffany Ford, Tanisha Hill-Jarrett, César Higgins Tejera, Lisa Barnes, Jennifer Manly

**Affiliations:** Columbia University, New York City, New York, United States; Columbia Mailman School of Public Health, New York City, New York, United States; Johns Hopkins University, Baltimore, Maryland, United States; University of Illinois at Chicago, Chicago, Illinois, United States; University of California, San Francisco, San Francisco, California, United States; University of Michigan, Ann Arbor, Michigan, United States; Rush University Medical Center, Chicago, Illinois, United States; Columbia University Irving Medical Center, New York City, New York, United States

## Abstract

Structural racism may drive the constellation of determinants that increase racialized disparities in cognitive function. The 1994 Crime Bill is a decades-old federal policy that established an infrastructure in which local governments are incentivized to fund prisons, increase average prison time, and implement adverse policing quotas. Foundational research suggests structural racism via high-contact policing and incarceration for persons racialized as Black, is associated with anxiety and depressed mood among, influencing cognitive function. Minimal research explores whether the intended beneficiaries of structural racism, persons racialized as White, have truly benefitted. We used US county-level incarceration data to compute the Black-White difference in jail and prison population rates between 1995-2005. These data were averaged by decade and linked by county to individual-level cognitive data for participants racialized as White (>50 years) in the Health and Retirement Study (N=13,016). Cognitive performance and cognitive change over time was assessed using a 27-item global cognitive score from the modified Telephone Interview for Cognitive Status for biannual years between 2006-20. A mixed effects model estimated the association of a one-unit change in county-level incarceration on cognitive performance. Greater 10-year Black-White disparities in the jail population were associated with greater cognitive performance among participants racialized as White one year later (b=0.13, 95% CI: 0.02, 0.25). Findings suggest structural racism via disproportionate high-contact policing and incarceration of persons racialized as Black is linked to better cognitive function for older adults racialized as White.

